# ^1^H^N^, ^13^C, and ^15^N resonance assignments of the *Clostridioides difficile* receptor binding domain 2 (CDTb, residues 757–876)

**DOI:** 10.1007/s12104-020-09979-y

**Published:** 2020-10-09

**Authors:** Mary E. Cook, Kristen M. Varney, Raquel Godoy-Ruiz, David J. Weber

**Affiliations:** Department of Biochemistry and Molecular Biology, Center for Biomolecular Therapeutics (CBT), University of Maryland School of Medicine, 108 N. Greene St., Baltimore, MD 21201 USA

**Keywords:** *Clostridioides difficile*, CDI, CDTb, Binary toxin

## Abstract

*Clostridioides difficile* is a bacterial pathogen responsible for the majority of nosocomial infections in the developed world. *C. difficile* infection (CDI) is difficult to treat in many cases because hypervirulent strains have evolved that contain a third toxin, termed the *C. difficile* toxin (CDT), in addition to the two enterotoxins TcdA and TcdB. CDT is a binary toxin comprised of an enzymatic, ADP-ribosyltransferase (ART) toxin component, CDTa, and a pore-forming or delivery subunit, CDTb. In the absence of CDTa, CDTb assembles into two distinct di-heptameric states, a symmetric and an asymmetric form with both states having two surface-accessible host cell receptor-binding domains, termed RBD1 and RBD2. RBD1 has a unique amino acid sequence, when aligned to other well-studied binary toxins (i.e., anthrax), and it contains a novel Ca^2+^-binding site important for CDTb stability. The other receptor binding domain, RBD2, is critically important for CDT toxicity, and a domain such as this is missing altogether in other binary toxins and shows further that CDT is unique when compared to other binary toxins. In this study, the ^1^H, ^13^C, and ^15^N backbone and sidechain resonances of the 120 amino acid RBD2 domain of CDTb (residues 757–876) were assigned sequence-specifically and provide a framework for future NMR-based drug discovery studies directed towards targeting the most virulent strains of CDI.

## Biological context

*Clostridioides difficile* infection (CDI) is caused by a spore-forming, Gram-positive bacterium, and it is the most commonly reported nosocomial infection in the world, accounting for 12% of all hospital-borne infections (Gerding 2015). Prior to the emergence of hypervirulent strains early in the twenty-first century, *C. difficile* strains produced only two large enterotoxins, TcdA and TcdB, which inhibit signaling pathways by glucosylating small GTPases. Whereas, hypervirulent strains emerging more recently, such as the NAP1 epidemic strain, encode TcdA/TcdB plus additional virulence factors, most notably a third toxin termed the *C. difficile* toxin (CDT) (Perelle et al. 1997). CDT is a binary toxin that kills host cells by covalent modification of essential intracellular regulators of host cell function, including G-actin. While drug options are becoming available to target the large clostridial toxins in CDI, TcdA/TcdB (Yang et al. 2015), there is nothing approved by the FDA to target CDT (Secore et al. 2017). To address this unmet medical need, potent and selective CDT inhibitors are needed to provide treatment option(s) for patients infected with the most serious hypervirulent CDT-containing strains of CDI.

CDT has an enzymatic subunit, CDTa (47.4 kDa), with ribosyltransferase activity, and a pore-forming delivery subunit, termed CDTb (74 kDa). Fully active CDT associates in a 1:7 ratio of CDTa to CDTb subunits (Goorhuis et al. 2008; Loo et al. 2005; McDonald et al. 2005; Rupnik 2008; Stewart et al. 2013; Geric et al. 2004). Prior to cellular entry via endosomes (Hale et al. 2004; Nagahama et al. 2000, 2004; Gibert et al. 2011), the binary toxin complex associates with membrane-bound host cell receptor(s), such as the lipolysis-stimulated lipoprotein receptor (LSR) and/or CD44 (Wigelsworth et al. 2012; Papatheodorou et al. 2011; Fagan-Solis et al. 2014; Hiramatsu et al. 2017). CDT enters the host cell via endosomes with the low pH in endosomes triggering CDTa translocation into the cytoplasm via the pore-forming entity, CDTb (Bachmeyer et al. 2001; Blocker et al. 2003; Haug et al. 2003; Knapp et al. 2002; Krantz et al. 2004, 2005, 2006; Lang et al. 2008; Schmid et al. 1994; Knapp et al. 2016). Upon delivery of the catalytically active CDTa enzyme into the host cell cytoplasm, ADP-ribosylation of G-actin occurs rapidly at Arg177 (Sundriyal et al. 2009). Modified G-actin leads to F-actin filament dissociation (Gulke et al. 2001), destruction of the cytoskeleton, increased microtubule protrusions, accelerated bacterial adhesion, and a “*death spiral*” for mammalian host cells (Gerding et al. 2014; Benz 2017; Barth 2017). Of interest here is the host-cell binding component of CDT, CDTb. Recent structural studies of CDTb showed that it exists in two states, either as an asymmetric or a symmetric di-heptamer, in the absence of CDTa (Gerding et al. 2014; Xu et al. 2020). The asymmetric form, ^Asym^CDTb, has one of its two heptameric components folding into a seven-stranded beta-barrel with an internal cavity that is reminiscent of the anthrax protective antigen (PA) (Akkaladevi et al. 2013). Although, when compared to other binary toxins, CDTb was found to have two distinct host cell receptor-binding domains, RBD1 and RBD2, rather than just a single domain. In addition, RBD1 lacks sequence homology to any other known toxin and contains a novel calcium-binding site needed for protein stability (Xu et al. 2020). Whereas, the RBD2 domain is absent altogether in other well-studied binary toxins, including anthrax. Importantly, RBD2 was shown to provide a potent dominant negative effect for host cell toxicity when isolated (residues 757–876) indicating its important functional role within hypervirulent CDI (Xu et al. 2020). Therefore, with these data in hand, the sequence-specific backbone and sidechain resonance assignments of RBD2 were completed as a first step towards designing RBD2 inhibitors using NMR-based methods, which will be important for the longer-term goal of targeting toxicities associated with hypervirulent *Clostridioides difficile* that produces CDT.

## Methods and experiments

### Protein expression and purification

As previously described (Xu et al. 2020), DNA encoding the RBD2 binding domain of CDTb (residues 757–876) was codon optimized and engineered into pET21 expression plasmid along with a His-tag at its N-terminus, as was needed for solubility and purification purposes (TOPGene Technologies). The RBD2-containing expression plasmid was transformed into the *E. coli* BLR(DE3) cells, and large-scale protein over-production was performed using 2 L of defined [^15^N, ^13^C]-labeled MOPS media, containing 1 g/L of ^15^N-labeled NH4Cl and 2.5 g/L of ^13^C-labeled D-Glucose per liter of media. IPTG (0.5 mM) was added to the media when the A_600_ cell density reading was between 0.7 and 0.8 O.D., at which time the cells were grown for an additional 18 h at 25 °C. At the end of the growth period, the cells were centrifuged for 15 min at 4000 r.p.m, and the pellets were weighed and resuspended in a lysis buffer containing 50 mM Tris–HCl, pH 8.0, 500 mM NaCl, 10 mM BME such that 3 mL of lysis buffer were added per gram of wet pellet. Prior to sonication, the cell lysate was incubated at 4 °C for 1 h with DNase, 10 mM CaCl_2_ and 10 mM MgCl_2_. Three cycles of sonication at 50% power amplitude were performed next and followed by centrifugation at 15,000 r.p.m for 45 min at 4 °C to remove cell debris. The clear supernatant was filtered and loaded onto a previously equilibrated HiPrep 16/60 IMAC column with buffer A (20 mM Tris–HCl, pH 8.0, 300 mM NaCl, 10 mM BME). Upon addition of buffer B (buffer A plus 1 M imidazole) to the column, the RBD2 construct was eluted at ~ 150 mM imidazole into 5 mL fractions and analyzed by SDS-PAGE. The RBD2-containing fractions were pooled, concentrated, and injected onto a Superdex S200-PG size exclusion column previously equilibrated with 15 mM HEPES buffer, pH 7.0, 150 mM NaCl, and 0.5 mM DTT. Fractions containing the RBD2 construct were eluted from the S200 column and shown to be pure via SDS- and native-PAGE (> 99%). The yield of the RBD2 domain was typically > 50 mg of purified protein per liter of bacterial cell culture, and its concentration was adjusted to ~ 1 mM, aliquoted, and stored at − 80 °C, prior to preparing NMR samples.

### NMR spectroscopy

**Fig. 1:**
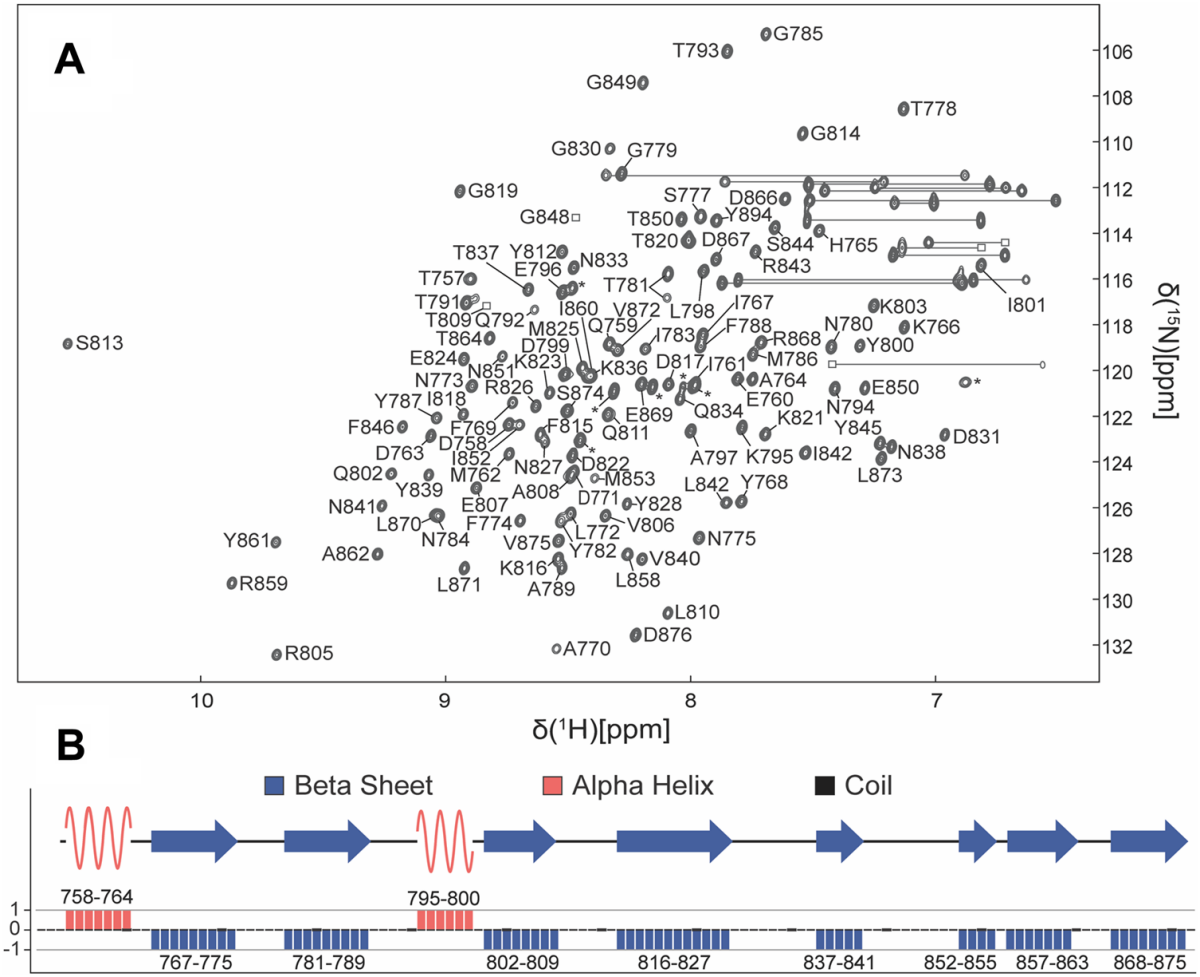
Resonance assignments and secondary structure of the receptor binding domain 2 (RBD2) domain of CDTb. **a** The 2D ^1^H,^15^N-edited HSQC spectrum of RBD2 (residues 757–876) recorded on a Bruker 950 MHz spectrometer at pH 7.0 and 25 °C. Residue type and number indicate assignments from backbone amide H^N^ correlations. Horizontal lines are illustrated for ^1^H-^15^N correlations arising from sidechain asparagine (Asn) and glutamine (Gln) residues, and correlations labeled with an asterisk (*) arise from residues within the His-tag region of the RBD2 construct. **b** The secondary structure of RBD2 predicted from the chemical shift index method is illustrated and consists of 8 beta strands (β1, I767-N775; β2, T781-A789; β3, Q802-T809; β4, K816-N827; β5, T837-N841; β6, I852-Y855; β7, K857-I863; β8, R868-V875) shown with blue arrows and 2 alpha helices (α1, D758-A764; α2, K795-Y800) highlighted in red. The remaining regions of RBD2 are predicted to exist as random coil and are shown with a black line in the secondary structure representation

NMR data was collected at 25 °C on Bruker 950 MHz, 800 MHz, and 600 MHz spectrometers. The NMR samples all contained 0.5 mM of the RBD2 domain, 15 mM HEPES buffer, pH 7.0, and 150 mM NaCl in 90% H_2_O and 10% D_2_O. The NMR sample was of high-quality as judged by a 2D ^1^ H,^15^ N-edited HSQC spectrum shown together with sequence-specific resonance assignments (Fig. 1a). The observable backbone and sidechain ^1^H, ^13^C, and ^15^N resonances were assigned via pairwise comparison of inter- and intra-residue ^13^ Cα, ^13^Cβ and ^13^ C´ chemical shift values from 3D HNCA, HN(CO)CA, HNCACB – CB optimized, CACB(CO)NH, HC(CO)NH, C(CO)NH, and HNCO experiments (Grzesiek 1992; Kay et al. 1994; Wittekind 1993; Montelione et al. 1992). The NMR data were processed with NMRPipe and analyzed using CcpNMR software (Delaglio et al. 1995; Vranken et al. 2005), and the secondary structure prediction was achieved using a Chemical Shift Index (CSI) algorithm on the CSI 2.0 web server (Berjanskii 2005). All proton chemical shifts were referenced to external trimethylsilyl propanoic acid (TSP) at 25 °C (0.00 ppm) with respect to residual H_2_O (4.698 ppm). The ^15^N and ^13^C chemical shift values reported were indirectly referenced using zero-point frequency ratios of 0.101329118 and 0.251449530, respectively (Wishart et al. 1995).

### Extent of assignment and data deposition

Sequence-specific resonance assignments shown in Fig. 1a were determined unambiguously using heteronuclear multidimensional NMR methods for 110 out of the 115 possible H^N^–^15^N correlations (~ 96%) of the receptor-binding domain-2 (RBD2) of CDTb. Of those 110 correlations, 100% of the CA, 95% of the CB, and 94% of the CO chemical shifts were determined. It was also possible to assign 7 of the 12 residues in the His-tag, which are labeled with an asterisk (*) in Fig. 1. The 120-residue RBD2 domain has five proline residues (Pro776, Pro790, Pro832, Pro835, and Pro865) that do not provide ^1^H–^H^N correlations. Four of the other five residues that do not appear in the 2D ^1^ H,^15^ N-edited HSQC spectrum reside in a small stretch of sequence in the C-terminal region of RBD2 (Thr854, Tyr855, Lys856, Lys857), and the fifth missing correlation was for residue Leu829. It is likely that non-proline missing correlations were the result of conformational averaging occurring on the chemical shift timescale. Two residues, Thr781 and Ileu783, were each found to have two H_N_ correlations having different ^1^H and ^15^N chemical shift values with varying intensities (~ 80:20), but the chemical shift values for their respective pairs of inter- and intra-residue carbon correlations to carbon (i.e. HNCA, HNCACB, etc.) were identical. While it is possible that peak doubling such as this could arise from a cis-trans proline isomerization, on the slow chemical shift timescale, a more likely explanation is that the sidechain of Tyr782 has two slowly exchanging conformational states. Nonetheless, providing a foolproof conclusion to this question of doubling is beyond the scope of this assignment note, and requires additional experimentation that will be reported elsewhere. The chemical shift values assigned for RBD2 were used next as input for a chemical shift index (CSI) algorithm to map the secondary structure of RBD2. As shown in Fig. 1b, the secondary structure from CSI analyses predicts that the RBD domain has eight beta strands (I767-N775; T781-A789; Q802-T809; K816-N827; T837-N841; I852-Y855; K857-I863; R868-V875) and 2 alpha helices (D758-A764; K795-Y800), which is fully consistent with the X-ray and cryoEM structures reported previously (Xu et al. 2020). In summary, the chemical shift values for backbone and sidechain resonances of RBD2 obtained here were deposited in the Biological Magnetic Resonance Bank database (http://www.bmrb.wisc.edu) under accession number 28,131, and these data will be important for next-stage NMR studies that map RBD2 biomolecular interactions and for developing inhibitors targeting CDTb.
